# Beyond Apical Ballooning: A Rare Focal Takotsubo Syndrome Variant Following Interscalene Block-Associated Airway Obstruction

**DOI:** 10.7759/cureus.113007

**Published:** 2026-07-20

**Authors:** Gonzalo J Martinez-Ruiz, Gerardo E Martinez-Arroyo, Frankie Alvarado-Abraham, Jonathan Cordero-Jimenez, Jorge J Ballester-Maldonado

**Affiliations:** 1 Internal Medicine, Universidad Central del Caribe (UCC) School of Medicine, Bayamón, USA; 2 Critical Care Medicine, Hospital Pavía Arecibo, Arecibo, USA; 3 Interventional Cardiology, Hospital Pavía Arecibo, Arecibo, USA; 4 Cardiology, Hospital Pavía Arecibo, Arecibo, USA

**Keywords:** cardiomyopathy, coronary angiography, echocardiography, myocardial infarction, takotsubo syndrome

## Abstract

Takotsubo syndrome (TTS) is a transient stress-induced cardiomyopathy that typically presents with apical ballooning; focal variants are rare and may closely mimic acute coronary syndromes. We present the case of a 74-year-old woman who developed acute hypoxemic respiratory failure secondary to vocal cord and phrenic nerve paralysis following an interscalene block. During hospitalization, she exhibited isolated troponin elevation without chest pain or electrocardiographic changes. Coronary angiography demonstrated nonobstructive coronary arteries, while ventriculography revealed focal lateral wall hypokinesis consistent with focal TTS. Initial management included full acute coronary syndrome therapy, which was subsequently de-escalated after the diagnosis was established. Acute airway obstruction is an uncommon trigger, underscoring the role of catecholamine-mediated myocardial stunning and the importance of early recognition to avoid unnecessary interventions and guide appropriate management.

## Introduction

Takotsubo syndrome (TTS), also known as stress-induced cardiomyopathy, is an acute and usually reversible syndrome characterized by transient left ventricular systolic dysfunction that occurs in the absence of obstructive coronary artery disease sufficient to explain the extent of myocardial dysfunction [1]. Although it classically presents with apical ballooning, several anatomic variants have been described, including midventricular, basal (reverse), and focal forms. Focal TTS is particularly uncommon, accounting for approximately 1.5% of reported cases, and frequently mimics acute coronary syndrome (ACS) because the regional wall-motion abnormality may correspond to the territory of a single coronary artery [1-3].

The exact pathophysiology remains incompletely understood, but excessive sympathetic activation with catecholamine-mediated myocardial stunning is considered the central mechanism. Physical stressors, including acute respiratory failure, neurological disorders, and critical illness, have increasingly been recognized as important precipitants and are often associated with more severe clinical presentations than emotional triggers [1,4].

We report a case of focal TTS occurring after acute hypoxemic respiratory failure caused by bilateral vocal cord paralysis following an ultrasound-guided interscalene brachial plexus block (ISB). The case highlights an unusual trigger, the diagnostic challenges posed by focal TTS in the absence of chest pain or ischemic electrocardiographic changes, the distinctive echocardiographic wall-motion pattern of the focal variant, and the importance of multimodality cardiac imaging in differentiating stress cardiomyopathy from ACS.

## Case presentation

**Figure 1:**
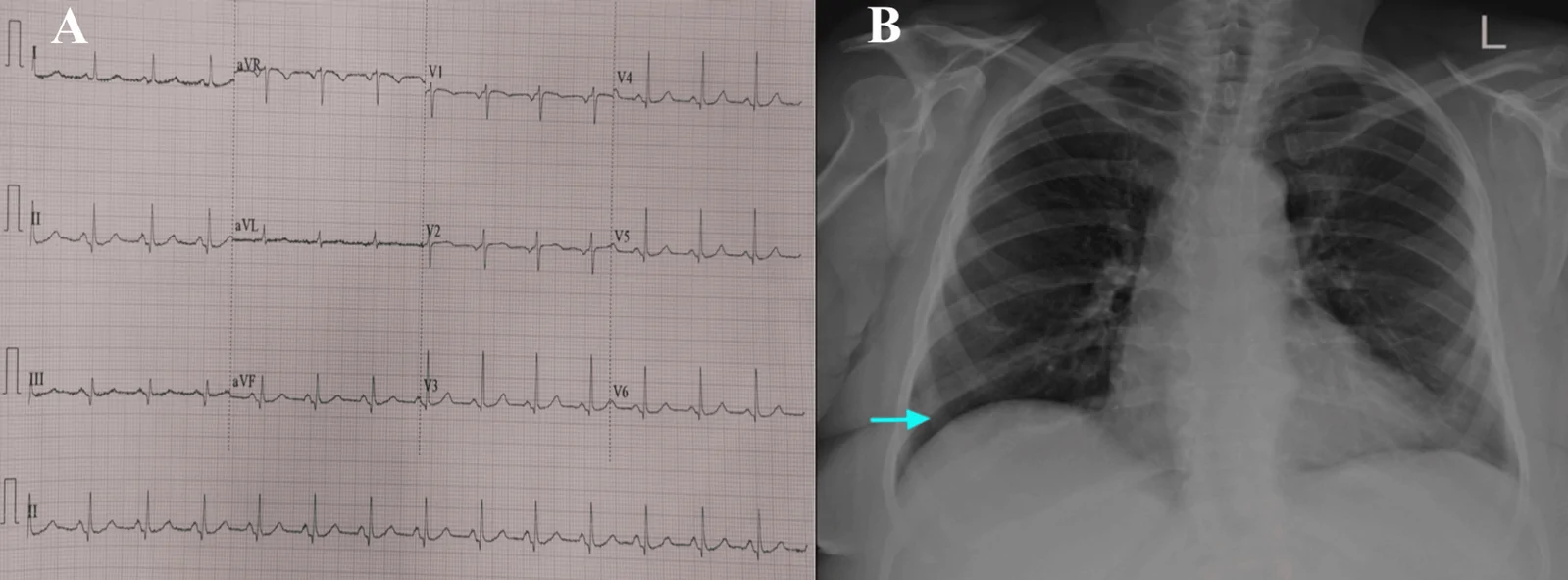
Preoperative evaluation Panel A: Preoperative 12-lead electrocardiogram obtained one day before the procedure, demonstrating normal sinus rhythm with a normal QRS axis. No significant ST-segment or T-wave abnormalities are present, and there is no electrocardiographic evidence of acute myocardial ischemia. This study served as the baseline electrocardiogram prior to the onset of postoperative respiratory failure and subsequent cardiac complications. Panel B: Preoperative chest radiograph demonstrating a normal right hemidiaphragm position (blue arrow), clear and well-expanded lungs, and a cardiomediastinal silhouette within normal size limits. No pleural effusion, pulmonary edema, or other acute cardiopulmonary abnormalities are identified. This image served as the baseline chest radiograph before the development of phrenic nerve paralysis and respiratory failure.

A 74-year-old woman with a past medical history significant for hypertension, type 2 diabetes mellitus, hyperlipidemia, anxiety disorder, obesity, and osteoarthritis underwent an elective right shoulder arthroscopy under ultrasound-guided ISB. Preoperative evaluation demonstrated low perioperative risk, with normal electrocardiogram findings (Figure 1A), chest imaging (Figure 1B), and preserved functional capacity.

Within hours post-procedure, she developed progressive dyspnea and severe hypoxemia refractory to supplemental oxygen. On arrival at the telemetry unit, she was in marked respiratory distress, non-verbal, and communicating only through gestures. Her condition rapidly deteriorated, with oxygen saturation declining into the low 60s despite high-flow oxygen and bag-valve-mask ventilation. Emergent endotracheal intubation was performed.

**Figure 2:**
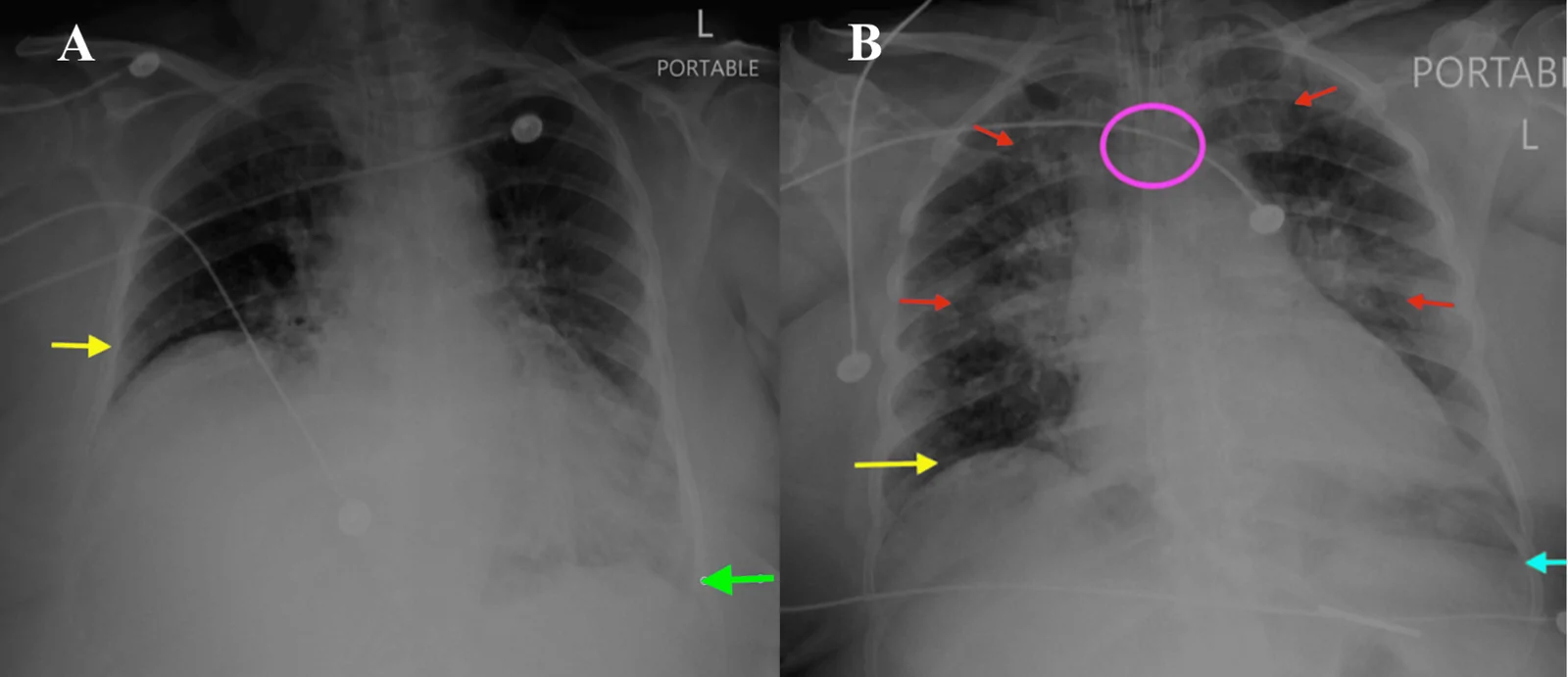
Postoperative chest radiographs Panel A: Postoperative chest radiograph demonstrating acute elevation of the right hemidiaphragm (yellow arrow) with a normally positioned left hemidiaphragm (green arrow), consistent with right phrenic nerve palsy following an interscalene brachial plexus block. The lungs remain clear without focal air-space consolidation, pleural effusion, or pneumothorax. This finding was new compared with the preoperative chest radiograph (Figure 1B) and raised concern for diaphragmatic dysfunction. Panel B: Post-intubation chest radiograph demonstrating bilateral pulmonary edema (red arrows), persistent elevation of the right hemidiaphragm (yellow arrow), a normally positioned left hemidiaphragm (blue arrow), and an appropriately positioned endotracheal tube (purple circle). These findings are consistent with negative-pressure pulmonary edema secondary to acute upper airway obstruction and respiratory failure.

Direct laryngoscopy revealed bilaterally adducted vocal cords without edema, consistent with upper airway obstruction. Pre-intubation imaging demonstrated an elevated right hemidiaphragm, suggestive of phrenic nerve paralysis (Figure 2A). Post-intubation chest X-ray (CXR) revealed bilateral pulmonary edema consistent with negative-pressure pulmonary edema (Figure 2B).

During ICU care, the patient remained hemodynamically stable. However, laboratory evaluation demonstrated elevated cardiac biomarkers (Table 1). Due to concern for ACS, a cardiology consultation was obtained. The differential diagnosis for this patient's acute respiratory failure, elevated cardiac biomarkers, and evidence of myocardial dysfunction included ACS, type 2 myocardial infarction secondary to severe hypoxemia and physiological stress, TTS, myocarditis, and pulmonary embolism. Given the marked troponin elevation and newly identified left ventricular systolic dysfunction, distinguishing among these diagnoses required multimodality cardiac and pulmonary evaluation.

**Table 1:** Laboratory findings Serial high-sensitivity (HS) troponin measurements demonstrated a marked rise from an initial normal value (20.5 pg/mL) to a peak of 3,157.6 pg/mL, followed by a downward trend, indicating acute myocardial injury. CPK-MB was mildly elevated, supporting myocardial damage. BNP was elevated, consistent with acute left ventricular dysfunction and heart failure. BNP: brain natriuretic peptide; CPK-MB: creatine phosphokinase-MB

Laboratory test	Result	Reference value	Units
HS troponin #1, 0 hours	20.5	0.0-58.9	pg/mL
HS troponin #2, 8 hours	3157.6 - High	0.0-58.9	pg/mL
HS troponin #3, 16 hours	2405.5 - High	0.0-58.9	pg/mL
CPK-MB	13.60 - High	0.50-3.60	ng/mL
D-dimer	0.29	0.19-0.49	mg/L
BNP	472 - High	0.0-100.0	pg/mL

Investigations

**Figure 3:**
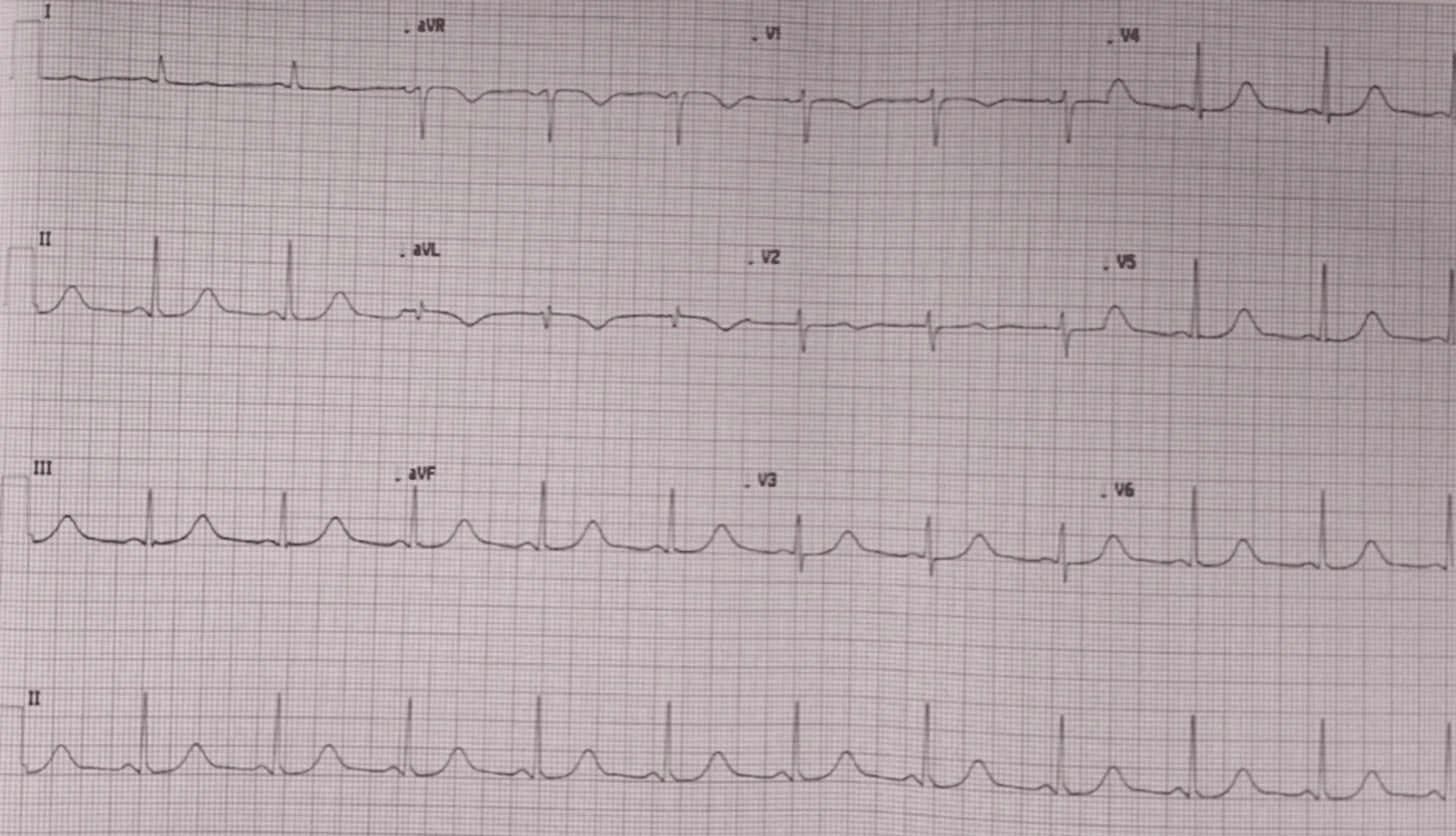
Repeat 12-lead electrocardiogram following clinical stabilization Repeat 12-lead electrocardiogram obtained following clinical stabilization demonstrates sinus rhythm and no acute ischemic ST-segment abnormalities. A small new Q wave with T-wave inversion in lead aVL is present. Although isolated and of uncertain clinical significance, this finding was not present on the preoperative electrocardiogram. Decreased QRS amplitude in lead aVL is also noted. No significant dynamic electrocardiographic changes were observed compared with the preoperative tracing.

**Figure 4:**
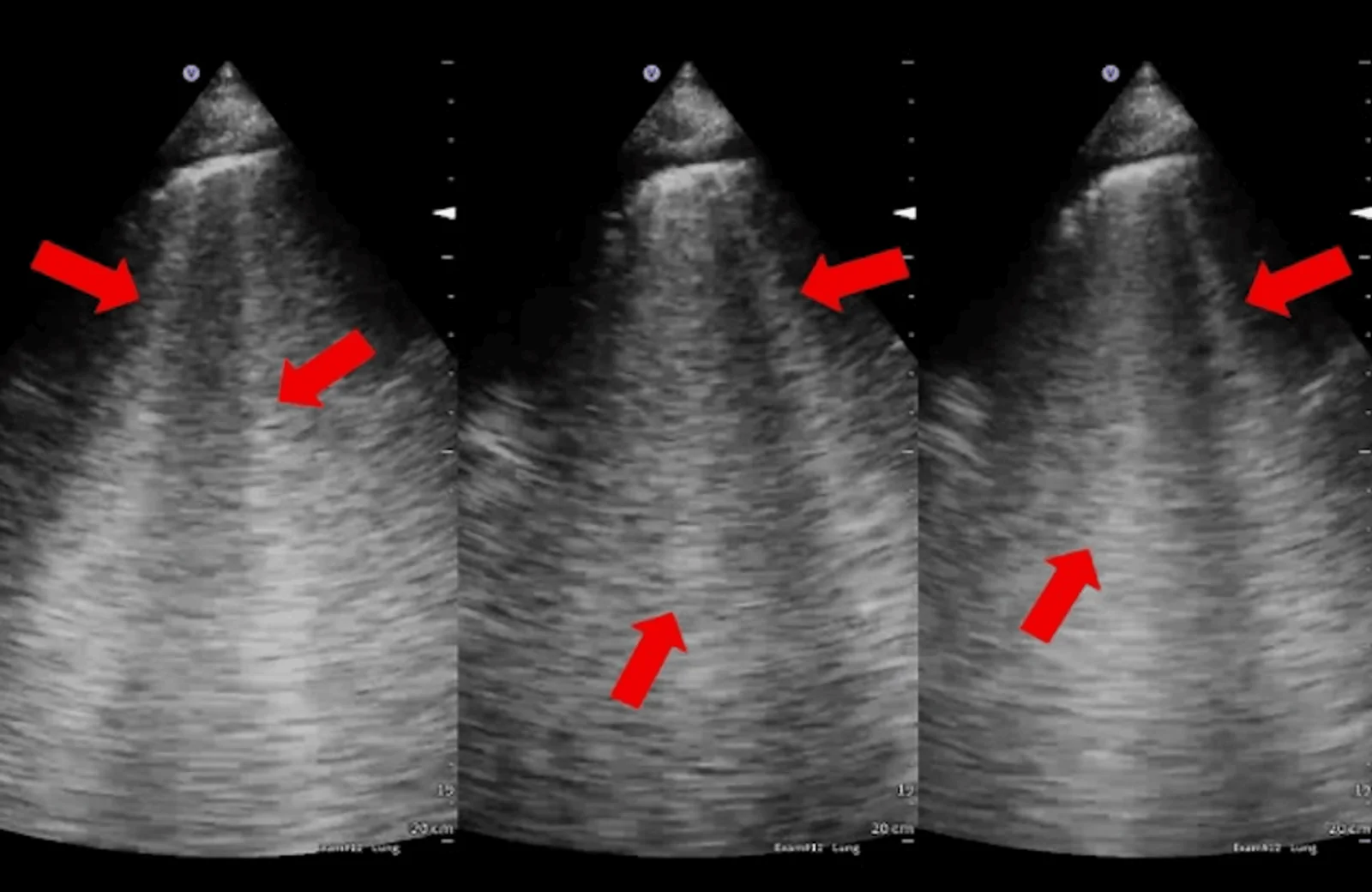
Point-of-care lung ultrasound Point-of-care lung ultrasound demonstrating diffuse bilateral B-lines across multiple scanning zones. More than three B-lines were identified per intercostal space in greater than three lung zones bilaterally, consistent with an alveolar-interstitial syndrome and pulmonary edema. Red arrows highlight representative B-lines. The diffuse distribution of B-lines supports the presence of pulmonary edema rather than a focal pulmonary process.

**Figure 5:**
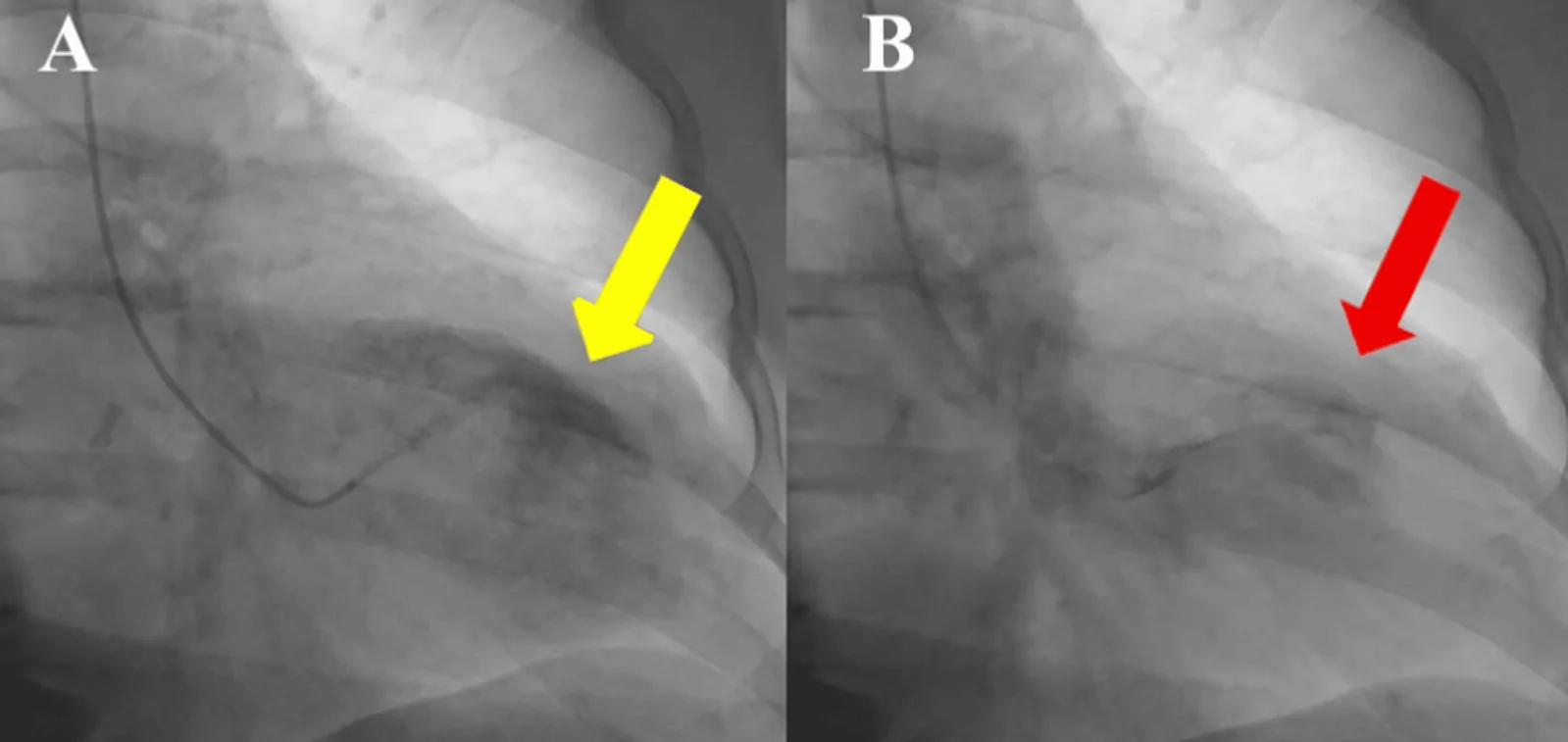
Left ventriculography Panel A: Left ventriculogram obtained during end-diastole demonstrating the mid-lateral left ventricular wall (yellow arrow), which serves as the reference segment for comparison with systolic contraction. Panel B: Left ventriculogram obtained during systole demonstrating focal hypokinesis of the mid-lateral left ventricular wall (red arrow), with preserved contractility of the remaining myocardial segments, consistent with the focal variant of Takotsubo syndrome. Comparison with the end-diastolic image (Panel A) highlights the reduced systolic excursion of the affected myocardial segment.

**Figure 6:**
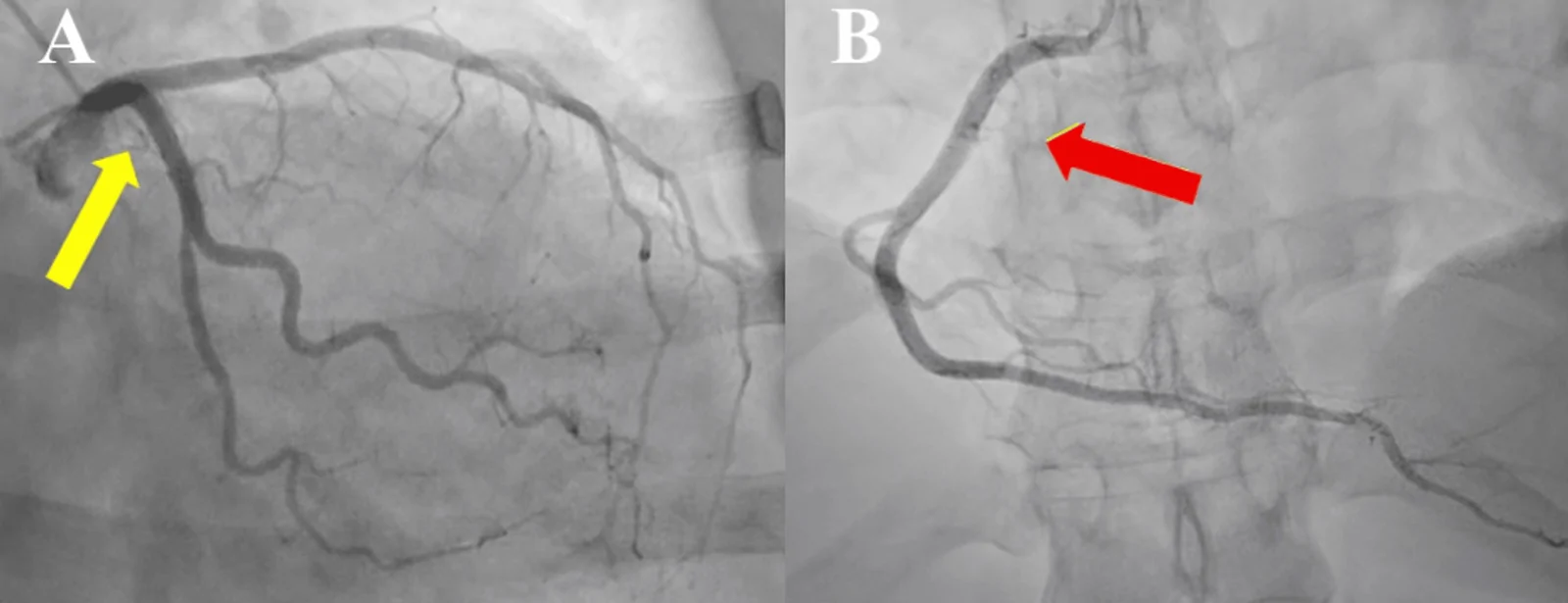
Coronary angiography Panel A: Coronary angiogram of the left coronary system demonstrating a patent left main coronary artery and its major branches without angiographically significant coronary artery disease. No flow-limiting stenosis or evidence of acute coronary occlusion is identified (yellow arrow). Panel B: Coronary angiogram of the right coronary artery demonstrating a patent vessel without angiographically significant stenosis or acute coronary occlusion (red arrow). The absence of obstructive coronary artery disease supports a nonischemic etiology for the patient's transient left ventricular systolic dysfunction.

Electrocardiograms demonstrated normal sinus rhythm without ischemic changes throughout the hospitalization (Figure 3). High-sensitivity troponin levels increased from 20.5 pg/mL to a peak of 3,157.6 pg/mL before declining to 2,405 pg/mL. Brain natriuretic peptide (BNP) was elevated at 472 pg/mL. Point-of-care ultrasound revealed diffuse bilateral B-lines consistent with pulmonary edema and showed no evidence of right ventricular strain (Figure 4). Transthoracic echocardiography revealed mildly reduced left ventricular systolic function with an estimated ejection fraction (EF) of 35-45%, accompanied by regional wall motion abnormalities involving the mid-lateral left ventricular wall, without significant valvular pathology (Video 1). Left ventriculography and coronary angiography were performed during the same cardiac catheterization procedure. Left ventriculography demonstrated focal hypokinesis of the mid-lateral wall with reduced systolic function (around 40%) (Figure 5 and Video 2), while coronary angiography demonstrated nonobstructive coronary arteries without evidence of significant coronary artery disease (Figure 6 and Video 3).

**Video 1:** Transthoracic echocardiography Transthoracic echocardiography demonstrating mildly reduced left ventricular systolic function with an estimated ejection fraction of 35%-45% and regional wall-motion abnormalities involving the lateral left ventricular wall. No hemodynamically significant valvular stenosis or regurgitation is identified. The observed wall-motion abnormality is consistent with the focal pattern of stress-induced cardiomyopathy and correlates with the findings observed on left ventriculography.

**Video 2:** Left ventriculography Left ventriculography demonstrating focal hypokinesis of the mid-lateral left ventricular wall with preservation of contractility in the remaining myocardial segments. No diffuse or apical ballooning pattern is observed. The localized wall-motion abnormality is characteristic of the focal variant of Takotsubo syndrome and correlates with the regional dysfunction identified on transthoracic echocardiography. The ventriculogram was obtained using a Judkins Left (JL) catheter with hand injection of contrast. Occasional ventricular ectopic beats are present during image acquisition but do not obscure visualization of the focal wall-motion abnormality.

**Video 3:** Coronary angiography Coronary angiography demonstrates nonobstructive coronary arteries without angiographically significant coronary artery disease. The left and right coronary systems are patent, with no evidence of flow-limiting stenosis, acute coronary occlusion, or angiographic features of plaque rupture. These findings effectively exclude obstructive coronary artery disease as the cause of the patient's left ventricular dysfunction and support the diagnosis of focal Takotsubo syndrome.

Management

Given the initial concern for ACS, the patient was treated with dual antiplatelet therapy, therapeutic anticoagulation, a high-intensity statin, an angiotensin receptor blocker, and a beta-blocker. Following angiographic confirmation of nonobstructive coronary arteries and ventriculographic findings consistent with Takotsubo cardiomyopathy, therapy was de-escalated to single antiplatelet therapy, and anticoagulation was reduced to prophylactic dosing. The beta-blocker and angiotensin receptor blocker were continued. Supportive management included mechanical ventilation, intravenous diuresis, and close hemodynamic and respiratory monitoring [5].

Outcome and follow-up

The patient demonstrated rapid clinical improvement following airway stabilization and supportive care. Pulmonary edema resolved with diuresis, and diaphragmatic function improved. She was successfully extubated within 48 hours.

According to the treating cardiologist, follow-up focused cardiac ultrasound demonstrated normalization of left ventricular systolic function (EF > 50%) with resolution of the previously observed regional wall-motion abnormalities, supporting the diagnosis of transient stress-induced cardiomyopathy. Although the follow-up images were unavailable for independent review, the patient remained clinically stable without recurrent symptoms.

## Discussion

This case highlights a rare presentation of the focal variant of TTS triggered by acute upper airway obstruction following ultrasound-guided ISB. Although TTS is classically associated with emotional or physical stressors, acute hypoxemic respiratory failure due to bilateral vocal cord paralysis and negative-pressure pulmonary edema is an exceptionally uncommon precipitating event [1,3]. Morphologic variants of TTS include apical (approximately 82%), midventricular (15%), basal (2%), and focal (1.5%) patterns, with the focal variant representing the rarest phenotype [3,5].

The diagnosis was particularly challenging because the clinical presentation lacked many of the features typically associated with TTS. Respiratory failure dominated the initial presentation, while chest pain and ischemic electrocardiographic abnormalities were absent. In this setting, myocardial injury could reasonably have been attributed to type 2 myocardial infarction secondary to profound hypoxemia and physiological stress. However, the marked troponin elevation together with regional wall-motion abnormalities on echocardiography prompted further cardiac evaluation. Compared with the classic apical variant, focal TTS may closely mimic ACS because the localized wall-motion abnormality often corresponds to the territory of a single coronary artery, making it particularly prone to misclassification as ischemic heart disease unless coronary angiography and careful ventriculographic assessment are performed [1,5].

Although our patient's presentation was otherwise highly consistent with the InterTAK Diagnostic Criteria, this case also highlights an important consideration when applying these criteria to the focal variant of TTS. Unlike the classic apical, midventricular, and basal forms, focal TTS may produce regional wall-motion abnormalities confined to the distribution of a single epicardial coronary artery, thereby reducing the discriminatory value of the criterion requiring wall-motion abnormalities beyond a single coronary territory [3]. Nevertheless, the diagnosis remained well supported by the identifiable physical trigger, absence of obstructive coronary artery disease, characteristic ventriculographic findings, modest biomarker elevation relative to the degree of ventricular dysfunction, and complete recovery of left ventricular systolic function on follow-up imaging. This case underscores the importance of integrating established diagnostic criteria with clinical judgment and multimodality cardiac imaging when evaluating uncommon morphologic variants of TTS [1-3].

Excessive sympathetic activation remains the most widely accepted mechanism underlying TTS. In our patient, acute airway obstruction likely produced a profound catecholamine surge triggered by severe hypoxemia and respiratory distress. Additionally, the markedly negative intrathoracic pressures generated during forceful inspiratory efforts against an obstructed airway, together with the development of negative-pressure pulmonary edema, may have further augmented sympathetic activation and contributed to myocardial stunning. Although only a few reports have described acute upper airway obstruction as a trigger of TTS, this mechanism is biologically plausible and consistent with the current understanding of stress-induced cardiomyopathy [2-4].

Because focal TTS closely resembles ACS, initial management should follow guideline-directed ACS therapy until obstructive coronary artery disease has been excluded [1,3]. Once the diagnosis is established, treatment is primarily supportive and directed toward resolution of the precipitating trigger, as recovery of ventricular function is expected in most patients [1,2,5]. In our patient, prompt airway stabilization and supportive care were accompanied by complete recovery of left ventricular systolic function, illustrating the favorable prognosis associated with timely diagnosis and appropriate management.

## Conclusions

This case expands the spectrum of recognized physical stressors associated with TTS by illustrating acute airway obstruction with hypoxemic respiratory failure as a potential trigger for focal stress cardiomyopathy. The atypical presentation, characterized by the absence of chest pain, minimal electrocardiographic abnormalities, and a focal pattern of ventricular dysfunction, highlights the diagnostic challenges posed by uncommon variants of the disease.

Furthermore, this case underscores the importance of timely multimodality cardiac imaging in patients with unexplained myocardial injury, as delayed evaluation may miss the transient ventricular abnormalities that define the syndrome. Recognition of atypical triggers and presentations is essential to improve diagnostic accuracy, guide appropriate management, and avoid misclassification as ischemic heart disease. As awareness of these uncommon manifestations grows, clinicians may be better equipped to identify TTS in critically ill patients and further refine our understanding of its diverse clinical phenotypes.
